# Biochar and compost effects on soil microbial communities and nitrogen induced respiration in turfgrass soils

**DOI:** 10.1371/journal.pone.0242209

**Published:** 2020-11-30

**Authors:** Muhammad Azeem, Lauren Hale, Jonathan Montgomery, David Crowley, Milton E. McGiffen

**Affiliations:** 1 Ningbo Urban Environment Observation and Research Station, Chinese Academy of Sciences, Ningbo, China; 2 Department of Environmental Sciences, University of California, Riverside, California, United States of America; 3 USDA, Agricultural Research Service, San Joaquin Valley Agricultural Sciences Center, Parlier, California, United States of America; 4 Department of Botany and Plant Sciences, University of California, Riverside, California, United States of America; RMIT University, AUSTRALIA

## Abstract

We examined the effect of a labile soil amendment, compost, and recalcitrant biochar on soil microbial community structure, diversity, and activity during turfgrass establishment. Two application rates of biochar (B1 at 12.5 t ha^-1^and B2 at 25 t ha^-1^), a 5 centimeter (cm) green waste compost treatment (CM) in top soil, a treatment with 12.5 t ha^-1^ biochar and 5 cm compost (B1+CM), and an unamended control (CK) treatment were prepared and seeded with tall fescue. Overall, results of phospholipid fatty acid analysis (PLFA) profiling and Illumina high-throughput sequencing of 16S rRNA genes amplified from soil DNA revealed significant shifts in microbial community structures in the compost amended soils whereas in biochar amended soils communities were more similar to the control, unamended soil. Similarly, increases in enzymatic rates (6–56%) and nitrogen-induced respiration (94%) were all largest in compost amended soils, with biochar amended soils exhibiting similar patterns to the control soils. Both biochar and compost amendments impacted microbial community structures and functions, but compost amendment, whether applied alone or co-applied with biochar, exhibited the strongest shifts in the microbial community metrics examined. Our results suggest application of compost to soils in need of microbiome change (reclamation projects) or biochar when the microbiome is functioning and long-term goals such as carbon sequestration are more desirable.

## Introduction

Amending soil with biochar increases carbon storage and can lead to changes in soil physical factors such as bulk density and water holding capacity; and chemical parameters including CEC, pH, and mineral nutrient content [1–3]. Biochar amendment may stimulate plant growth through enhanced microbial activity [4] and can affect microbial abundance, bacteria/fungi ratio and community structure [5–8].

Biochar induces shifts in soil microbial abundance and community composition [9, 10], and can influence N turnover [11, 12]. However, the results are often inconsistent [13, 14]. Most previous agricultural experiments used freshly generated biochar [15, 16], and the variable properties of fresh biochar may cause inconsistent results on plant and soil biological performances [17]. Biological modification of biochar, such as through co-composting or the action of common compost by-products such as humic and fulvic acids [18, 19] or adding plant growth promoting bacteria or fungi [20] have been suggested as means to achieve better agronomic performance. Biochar may also affect other organic amendments by stabilizing organic matter as it undergoes decomposition, leading to increased SOM levels and soil aggregate formation [21, 22]. The interior pores of charcoal particles could also serve as a protected habitat where beneficial bacteria and fungi can reside [20].

Turfgrass grown in arid regions faces problems of low organic carbon, poor soil fertility and water shortage. In water-limited ecosystems, managing soil carbon and the availability of soil water is essential for maintaining productivity and plant performance [23]. Several previous studies suggest that amending soil with biochar will increase soil carbon and water content [24, 25], but most of the previous turfgrass research evaluating the use of compost and biochar has been greenhouse experiments with turf grown in sand-based media; these papers often recommend further evaluation under field conditions [26–29]. The potential link between organic-amendment-induced shifts in microbial community composition and variances in soil N and C cycling function has yet to be systematically examined in turf soil, despite the high water and nutrient consumption of the 35,850 km^2^ of managed turfgrass in the United States [30].

Overall, studies indicate that biochar and organic substrates are likely to bring synergistic benefits to soil fertility. However, very few studies have examined if these benefits are maintained in the field, or their effects on soil microbial communities, enzyme actives and nitrogen induced carbon losses. We conducted field experiments to evaluate the effect of compost and biochar amendments on the turfgrass soil microbiome, including their effects on bacterial community biomass, structure, including fungal bacterial ratios, bacterial/archaeal diversity, and relative abundances of bacterial taxonomic groups associated with N cycling and soil C storage. Simultaneously we assayed microbial enzymatic activities and N-induced soil heterotrophic respiration, as well as bacterial and fungal specific soil respiration rates. From these results we address how biochar and compost organic amendments impact turf community structures and functions.

## Material and methods

### Site description

The turf plots were established at the Agricultural Experiment Station at the University of California, Riverside USA (33°58′32″N 117°19′52″W) on an Arlington sandy loam soil with pH of 8.34 and an average soil organic carbon (SOC) of less than 1%. The climate of the site is semiarid-Mediterranean, with hot and dry summers, mild and relatively wet winters. The summer daily 24-hr average temperatures are 32°C but frequently exceed 38°C. The annual rainfall is 264 mm with most of it occurring in the winter and early spring.

### Biochar and compost production and application

Biochar was derived from a yellow pine sawdust feedstock by pyrolysis at 350°C for 48 hours. Compost was produced from tree trimmings (leaves and branches) that were ground to pass through a 10 cm sieve, watered to 50% moisture, and placed in windrows 3 m in height. The windrows were thoroughly mixed every 3 days to redistribute particles for uniform composting and to maintain an internal temperature of 55°C, and water added to maintain 50% moisture. The process was repeated until stable temperature and CO_2_ evolution was reached 15 days after the windrows were formed. Physicochemical properties of the amendments are listed in Table 1.

**Table 1:** Basic characteristic of unamended soil, green waste compost and biochar (0–15 cm).

Parameter	Unamended Soil	Green Waste Compost	YP350 Biochar
pH	8.34	7.71	7.45
Electrical conductivity (EC) dS m^-1^	1.3	2.4	0.12
Total nitrogen (%)	1.1	0.67	0.33
Ammonia (NH_4_-N) (mg kg^-1^)	-	21	24
Nitrate (NO_3_-N) (mg kg^-1^)	4.2	< 1	1
Organic nitrogen (%)	-	0.67	0.33
Phosphorus (mg kg^-1^)	25	1300	122
Potassium (mg kg^-1^)	61	6100	1476
Organic Carbon (%)	0.46	38	75.6
Particle Size (cm)	_	11.7	0.04

Treatments included a control (CK) with no amendments, biochar at 12.5 t ha^-1^ (B1), biochar at 25 t ha^-1^ (B2), 5 cm green waste compost (CM), and biochar at 12.5 t ha^-1^ + 5 cm green waste compost (B1+CM). The amendments were uniformly incorporated into the upper 15 cm of soil. Each of four replicate plots for each treatment measured 3 x 3 m^2^ and were arrranged in a randomized block design. After incorporation of the soil amendments in April 2014, the plots were seeded with tall fescue in May 2014 and the turf was established to a dense cover through the first year.

### Soil sampling and physicochemical analysis

The plots were sampled in October 2014, 6 months after amendments were applied, as the turf was re-greening and undergoing active growth. Topsoil cores from the upper 0–15 cm depth were collected randomly with a 2.5 cm diameter probe at three points within each replicate plot, then composited. The samples were preserved in polythene bags and brought to the laboratory within a half hour after sampling. Root debris was removed. For physicochemical analysis the air-dried soil was ground to pass a 2 mm sieve before analysis and were stored and room temperature. For microbial analyses, fresh soil samples were kept at -80°C until DNA and PLFA extractions were performed. Gravimetric soil water content (SWC) was determined by the procedures of Gardner *et al*. [31]. In brief, soil samples were weighed, oven-dried (105°C), cooled (25°C) and then reweighed. The soil pH was analyzed in soil:water (1:5) suspension [32]. The soil total carbon (TC) and nitrogen (TN) contents of the biochar samples were determined using CNS on a Flash EA1112 analyzer (Thermo Scientific).

### Enzymes analysis

To quantify urease activity (UA), 5 g soil were placed in 100 mL flasks then 2.5 mL urea were added and soil was incubated for 2 hours (h) at 37°C. Then 50 mL KCl were added into the flasks and after 30 mins extracts were collected and treated with Na salicylate/NaOH+sodium dichloroisocyanide solution. Ammonium chloride was used for calibration curves with ammonium concentrations of 0, 1, 1.5, 2, 2.5 μg NH_4_-N mL^-1^. The sample filtrate and standard optical densities (OD) were determined at a wavelength of 690 nm [33] and was expressed as μg NH_4_-N g^-1^ dw 2 h^-1^. To measure dehydrogenase activity (DHA) the reduction of 2, 3, 5-triphenyltetrazolium chloride (TTC) into triphenyl formazan (TPF) was determined. After filtration, the OD of the soil extract was determined at 546 nm wavelength on a spectrophotometer. The DHA was measured as; TPF (μg mL^-1^) x 45/dwt/5 [33]. The β-glucosidase activity (BGA) was analyzed by colorimetric determination of BGA conversion of p-nitrophenyl-β-D-glucopiranoside to p-nitrophenol [34]. In brief, 1 g soil samples were incubated for 1 h (37°C) after the addition of p-nitrophenyl-β-D-glucopiranoside buffer having pH of 6.0 and toluene solution. After incubation, the BGA activity was analyzed by determining the yellow filtrate colorimetrically via reaction with 1mL 0.M CaCl_2_ and 4 mL tris (hydroxymethyl) aminomethane buffer (pH:12). The BGA was measured as μg p-nitrophenyl g^-1^ dry soil h^-1^.

### Extraction of phospholipid fatty acids (PLFAs) from soils

To extract soil PLFAs, 5 grams dry weight soil were placed in glass centrifuge bottles. In each tube, the methyl nonadecanoate fatty acid (19:0) was added as an internal standard [35]. In the first extraction, chloroform, methanol and phosphate buffer was added (1:2:0.8), while second extraction was carried out after 2 h via the addition of chloroform and buffer to achieve an ultimate ratio of 1:1:0.9 (chloroform: methanol: buffer). After 18 h, the organic phase was centrifuged and shifted to a test tube to evaporate solvent under nitrogen gas (N_2_) [36, 37].

### Methylation and formation of fatty acid methyl esters (FAMEs)

Lipid methylation was conducted using a methanol and toluene solution added to each sample in a 1:1 ratio followed by addition of KOH, samples were incubated in a water bath at 37°C for 15 minutes. After cooling to room temperature, 0.5 mL of acetic acid was added to each tube. For phase separation, 2 mL chloroform, then 2 mL chloroform-extracted deionized water (DI) were added in each sample, which separated by phase. The organic layer (bottom) was transferred to another tube and washed/ suspended/ separated with chloroform in several rounds. After this methylation step, PLFAs were transformed to FAMEs which were re-suspended in chloroform (5 mL). Chloroform was allowed to volatilize under N_2_ blow-down and samples were stored at –20°C, until suspension in hexane and analysis on a gas chromatographer (GC) HP 6980- Hewlett Packard; Wilmington, Del. with FID (Flame Ionization Detector) equipped with the HP 3365 column and Chem-Station and MIDI-Sherlock software. Markers used to profile the microbial community were; 15:0-ISO; 15:0-ANTEISO; 16:0-ISO; 17:0-ISO; 17:0-ANTEISO for Gram^+^, 16:1-w7c; 17:0-CYCLO for Gram^-^, 16:1-w5c; 18:3-w6c; 18:2-w6c for fungi, 16:1-w5c for arbuscular mycorrhizal fungi (AMF), and 16:00, 18:1-w9t Alcohol for *Pseudomonas* (PSU) [38].

### MiSeq analysis

To profile bacterial 16S rRNA genes using high throughput sequencing, the variable region (V4) of 16S rRNA genes were targeted using PCR primers 515f and 806r with a barcode on the forward primer. PCR reactions were prepared using the HotStarTaq Plus Master Kit (Qiagen, USA). The PCR conditions were; 3 minutes (94°C), followed by 28 cycles of 30 seconds at 94°C, 40 seconds at 53°C, and 1 minute at 72°C and a final elongation step for 5 minutes (72°C). To check the success of the relative intensity of bands and amplification, PCR products were run in a 2% agarose gel. On the basis of DNA concentrations and their molecular weight, multiple samples were pooled together (e.g., 100 samples) in equal proportions. After that, samples were purified using calibrated Ampure XP beads and PCR product was used to arrange a DNA library by following the Illumina TruSeq DNA library preparation protocol. Amplicon library preparation and ssequencing were performed at a commercial facility, M.R. DNA Lab, on the Illumina MiSeq platform following the manufacturer’s procedures (www.mrdnalab.com, Shallowater, TX, USA). The sequencing data was processed using M.R. DNA analysis pipeline. In brief, sequences were joined, depleted of barcodes, then sequences less than 150 base pairs and sequences with vague base calls were removed. Sequences were denoised and chimeras were removed. OTUs were defined by clustering 97% sequence identity. Final OTUs were taxonomically classified using BLASTn against a curated database derived from GreenGenes, RDPII, and NCBI [39]. Prior to analyses of alpha diversity and environmental correlations, the OTU table was rarefied to 11,590 reads per sample, which was sufficient to represent treatment trends based on rarefaction analysis.

### Selective inhibition and substrate induced respiration (SIR)

To determine the soil respiration, 15 g soil samples were taken in vials and three levels of nitrogen (urea) were applied i.e., 0 ppm (N0), 50 ppm (N50) and 100 ppm (N100) with four replications, while N0 was considered as a control. Glucose (5 mg g^-1^ soil) was added to each N treatment after N addition, soil moisture was brought to field capacity, and soil was incubated at room temperature. The initial measurement was done 1 h after the addition of nitrogen and glucose. The lids were not tightly closed before measurements, while before incubation (1 h), they were tightly closed to accumulate CO_2_ in the headspace. CO_2_ was analyzed by taking a gas sample in a syringe (30 ml), which was determined on EGM4-Analyzer, USA. To reduce the diurnal effects, readings were taken each day at the same time for 16 days. Depending on the bulk densities of each sample, the flux per hour (F = g·kg^-1^·h^-1^) was measured by using the formula [40];

$$F\;(mg\;{kg}^{-1}\;h^{-1})\;=\;\rho (\frac{{CO}_{2}(ppm)}{\Delta t})(\frac{V^{3}}{m})(\frac{K}{K+{}^{\circ}C})$$

Where,

P = density of gas at 1atm

V = volume of headspace

T = time in hour

M = Mass of dry soil

K = Temperature in kelvin

Fungal and bacterial respirations were determined using the selective-inhibition procedures [41]. The streptomycin (6 mg g^-1^ of soil) was used as a bacterial respiratory inhibitor while cycloheximide (13 mg g^-1^) was taken as a fungal respiratory inhibitor. Cycloheximide was applied to soil (15 g, dry-weight soil) 4 h prior to glucose addition (5 mg g^-1^ soil, powder), while streptomycin solution was added 0.5 h prior to glucose addition. The samples were incubated at 22°C and the respiration was determined after 4 h incubation with a CO_2_ analyzer.

### Statistical analysis

Two-way analysis of variance (ANOVA) was performed to analyze the differences among treatments for soil respiration rates, enzymatic activities, and physio-chemical properties. Time scales of N-induced respiration rates were analyzed by multifactorial analysis of variance (ANOVA) using RCBD. Multiple pairwise comparisons to attribute qualities significantly different based on treatments were performed post-hoc using the Holm- Šídák method. All ANOVAs and post-hoc tests at significance levels of p *≤* 0.05, and Two-tailed T-tests were performed using SigmaPlot 14.0. Canonical correspondence analysis (CCA) was used to correlate the soil properties, enzymes, PLFA, and nitrogen-induced respiration rates. To evaluate shifts in bacterial/archaeal community profiles and correlations to soil chemistry and amendment treatments, the 16S rRNA OTU table was rarefied to 11,590 reads per sample. Then, Mantel tests and Person and Spearman correlations were employed to compare microbial community data to activities, soil chemistry, and amendments, Nonmetric Multidimensional Scaling (NMDS) analyses based on Bray-Curtis dissimilarity matrices was used to evaluate β-diversity of microbial communities across treatments. Shannon diversity indices were calculated to estimate community α-diversity, where the Shannon index was defined as *H* = -∑ p_i_ log (b) p_i_, where p_i_ is the proportional abundance of species i and b is the base of the logarithm, in this case b was set to 2. Both NMDS analysis and Shannon index calculations were conducted in R packages Vegan and ampvis2 [42, 43]. Random Forest analyses were performed using class level taxonomic tables of 16S sequencing reads after removing classes that were present in less than 20% of the samples. The importance of each bacterial/ archaeal class in distinguishing the amended soils (e.g. contained biochar or contained compost) was determined across 500 trees using R package random Forest v4.6–14 [44].

## Results

### Effect of biochar on soil physicochemical properties

Physicochemical properties of the soil, green-waste compost and biochar are presented in Table 1 and the amendment effects on soil physicochemical properties are presented in Table 2. Compared to the control soil, soil moisture and TC were significantly enhanced in compost amended treatments. A significant decrease in soil pH was noticed in B2 and caused a 13% decrease compared to the control (Holm- Šídák test, P < 0.05). On the other hand, no significant effects on TN, total phosphorus (TP), and total potassium (TK) were observed from biochar treatments, while NO_3_ was significantly decreased with biochar addition. However, compost enhanced TN, TP, and TK concentrations. Altogether, co-use of biochar and compost had the greatest effect on soil nutrient and water contents, exhibiting chemical properties similar to the compost treatment, with the exception of TK, which were significantly higher.

**Table 2:** Biochar and compost effect on soil properties.

Treatments	pH	SWC (%)	TC (%)	TN (%)	NO_3_ (mg kg^-1^)	TP (mg kg^-1^)	TK (mg kg^-1^)
C	8.34±0.1 a	15.04±4 b	0.46±0.2 b	0.22±0.06 ab	4.33± 0.07 b	24±2 b	60±2 c
B1	7.55±0.1 a	21.93±8 ab	0.49±0.1 b	0.20±0.02 ab	3.34± 0.06 cd	28±1 b	62±2 c
B2	7.22±1.4 b	26.88±2 ab	0.55±0.1 b	0.16±0.02 b	2.71± 0.05 d	30±1 b	65±2 c
CM	7.86±0.6 a	28.22±1 a	1.33±0.3 a	0.25±0.07 ab	5.27± 0.06 a	47±6 a	78±7 b
B1+CM	7.82±0.8 a	27.50±9 a	1.14±0.3 a	0.29±0.09 a	3.94±0.60 bc	53±6 a	92±6 a
P-value (ANOVA)	0.377	0.016	0.001	0.008	0.001	0.000	0.000

Treatments include unamended control (CK), 12.5 t ha^-1^ biochar (B1), 25 t ha^-1^ biochar (B2), 5 centimeter (cm) green waste compost (CM), and 12.5 t ha-1 biochar and 5 cm compost (B1+CM). Different letters indicate values that are significantly different across treatments (Holm-Šídák, P ≤ 0.05).

### Microbial community structure, but not total biomass varied across treatments

Based on PLFA data biochar and compost application had non-significant effects on soil microbial biomass (Table 3). A low proportion of the soil biomass was attributed to fungi, averaging approximately 20%, with no significant differences across treatments. A small increase in the proportion of biomass characterized as Gram-positive bacteria was observed in the B2 treatment, compared to the control. A small decrease (but non-significant) in *Pseudomonas* biomass was also noticed in B2 compared to compost amended treatments, CM and B1+CM.

**Table 3:** PLFA-based profiles of soil microbial community under biochar and compost amendments.

Source	Total biomass	Bacteria	Fungi	BF Ratio	Gram +ive	Gram -ive	Pseudomonas	Mycorrhizae
C	68±2 a	55±3 a	12±3 a	0.22±0.06 a	5.9±0.9 ab	5.7±0.6 a	9.7±1.4 ab	7.8±2.7 a
B1	67±4 a	56±3 a	11±2 a	0.20±0.04 a	6.25±0.5 ab	5.6±0.4 a	9.4±1.5 ab	6.7±1.0 a
B2	66±2 a	55±3 a	11±1 a	0.19±0.02 a	6.7±1.2 a	5.3±0.3 a	8.2±0.6 b	5.9±1.2 a
CM	67±3 a	55±3 a	13±4 a	0.24±0.07 a	5.2±0.7 b	5.0±0.6 a	11.3±0.9 a	6.3±2.5 a
B1+CM	69±4 a	53±2 a	14±2 a	0.27±0.03 a	5.3±0.6 ab	5.00±0.4 a	10.9±0.5 ab	7.8±1.4 a
P-value (ANOVA)	0.741	0.677	0.301	0.249	0.146	0.124	0.022	0.596

Values are in nmols PLFA g^-1^ soil. Treatments include unamended control (CK), 12.5 t ha^-1^ biochar (B1), 25 t ha^-1^ biochar (B2), 5 centimeter (cm) green waste compost (CM), and 12.5 t ha^-1^ biochar and 5 cm compost (B1+CM). Different letters indicate values that are significantly different across treatments (Holm-Šídák, P ≤ 0.05).

**Fig 1:**
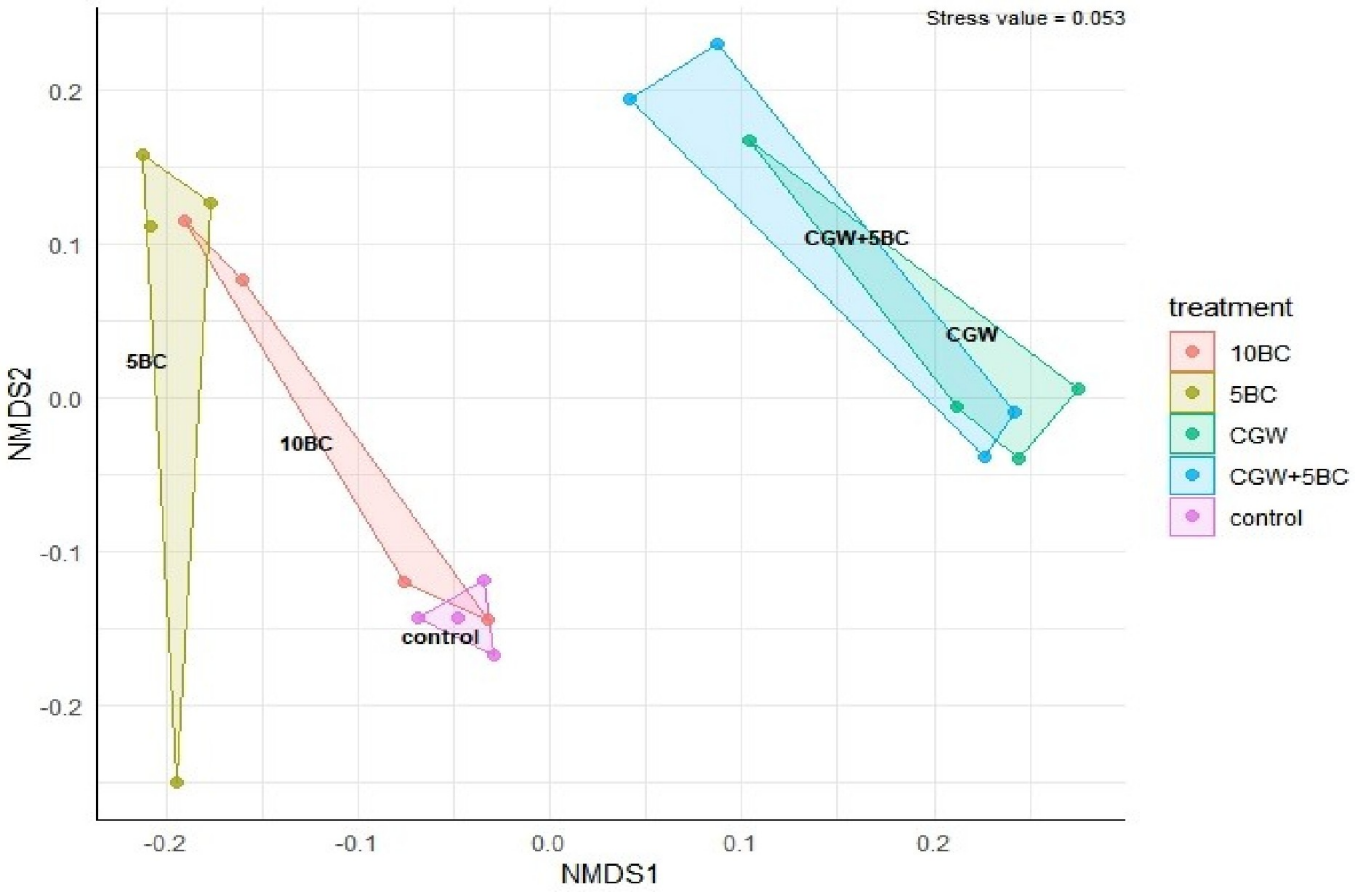
Nonmetric multidimensional scaling (NMDS) based on Bray-Curtis community dissimilarity revealed that bacterial/ archaeal community compositions in compost amended soils were most dissimilar to the unamended CK soil. Treatments include unamended control (CK), 12.5 t ha^-1^ biochar (B1), 25 t ha^-1^ biochar (B2), 5 centimeter (cm) green waste compost (CM), and 12.5 t ha^-1^ biochar and 5 cm compost (B1+CM).

**Fig 2:**
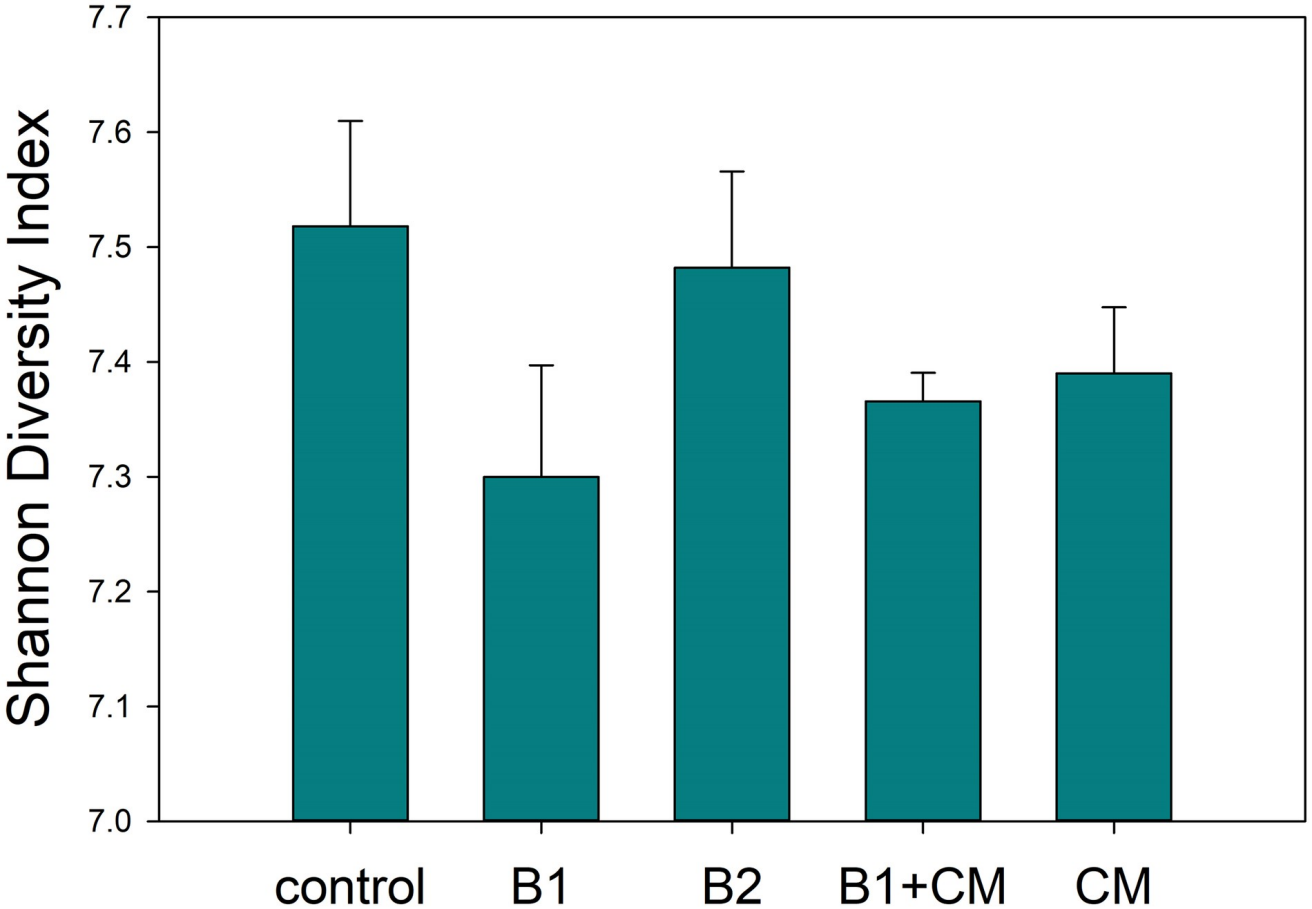
Bacterial/ archaeal community α-diversity based on Shannon indices indicated that the low biochar treatment (B1) significantly reduced community diversity compared to the control (Holm-Sidak, P < 0.05). Treatments include unamended control (CK), 12.5 t ha^-1^ biochar (B1), 25 t ha^-1^ biochar (B2), 5 centimeter (cm) green waste compost (CM), and 12.5 t ha^-1^ biochar and 5 cm compost (B1+CM).

Further investigation into bacterial/archaeal community taxonomic compositions was performed using 16S rRNA high-throughput sequencing. Microbial communities were most dissimilar from the control treatment when compost was added to turf plots (NMDS, Fig 1). The bacterial/ archaeal community alpha diversity was significantly lower in the B1 treatment than the control, but otherwise was not significantly impacted (Fig 2).

**Fig 3:**
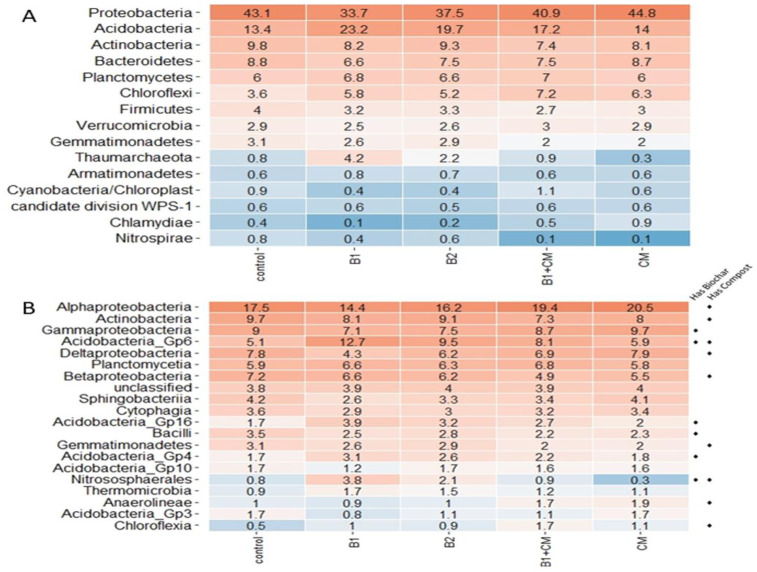
Heatmap depicting the dominant bacterial phyla (A) and classes (B) in the turfgrass plot soils. ♦ symbols identify classes that were important in delineating the soil treatments (presence or absence of biochar or compost) based on Random Forest analyses (%MSE in upper 25% range). Treatments include unamended control (CK), 12.5 t ha^-1^ biochar (B1), 25 t ha^-1^ biochar (B2), 5 centimeter (cm) green waste compost (CM), and 12.5 t ha^-1^ biochar and 5 cm compost (B1+CM).

On the phylum level, all amendments exhibited increased relative abundances of *Acidobacteria*, and *Choloroflexi* compared to the control plots (Fig 3A). However, *Actinobacteria*, *Firmicutes*, *Cyanobacteria*, and *Nitrospirae* phyla all exhibited lower relative abundances in amended plots compared to the control. The influence of biochar and compost differed in the shifting abundances of many phyla. For example, biochar application resulted in reduced *Proteobacteria*, *Bacteriodetes*, and *Chlamydiae* relative abundances, whereas compost with or without biochar resulted in their increased or unchanged relative abundances. Compost, on the other hand, exhibited decreased *Gemmatimonadetes* compared with the control. Class-level taxonomic classifications revealed lower relative abundances of *Actinobacteria*, *β-Proteobacteria*, *Bacilli*, and *Gemmatimonadetes* and higher relative abundances of *Acidobacteria* Gp6, Gp16, and Gp4, and *Chloroflexia* in all amended soils compared to the control (Fig 3B). Compared to the control biochar plots also exhibited reduced relative abundances of *α-Proteobacteria*, *γ- Proteobacteria*, *δ-Proteobacteria*, and *Spingobacteriia* and increased *Planctomycetia*, *Nitrososphaerales*, and *Chloroflexia*. Compost amended plots, with and without biochar exhibited increased relative abundances of *α-Proteobacteria*, and *Anaerolineae* compared to the control plots.

### Microbial activity was higher in soils receiving compost with and without biochar

**Fig 4:**
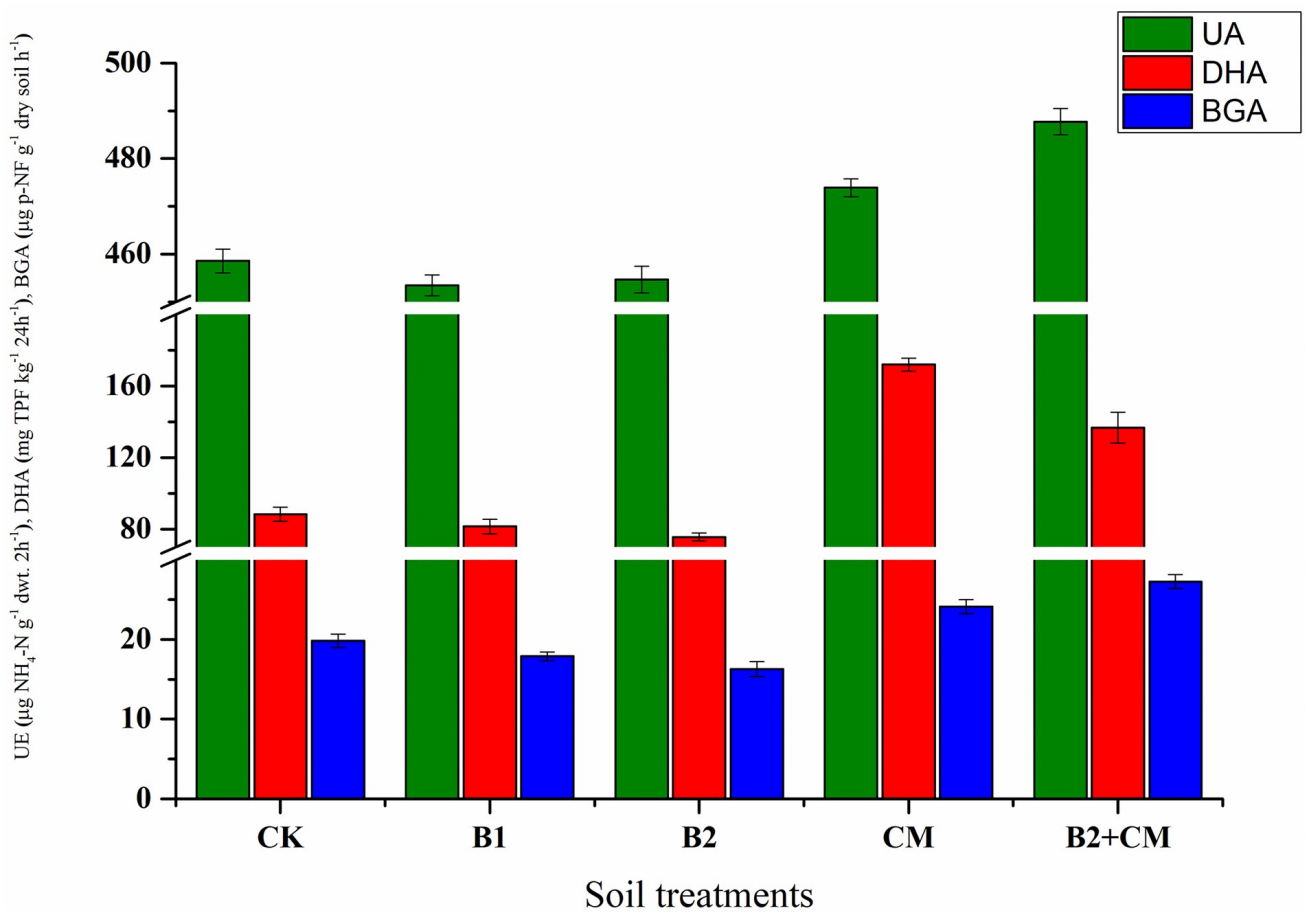
Urease (UA), dehydrogenase (DHA) and β-glucosidase activity (BGA) in soils was influenced by biochar and compost application (P < 0.05). Treatments include unamended control (CK), 12.5 t ha^-1^ biochar (B1), 25 t ha^-1^ biochar (B2), 5 centimeter (cm) green waste compost (CM), and 12.5 t ha^-1^ biochar and 5 cm compost (B1+CM).

Biochar application alone had a non-significant effect on microbial enzyme activity, while compost alone and with co-use of biochar significantly boosted all three enzyme activities (Fig 4). Treatment B1+CM exhibited 6, 54 and 54% increase in UA, DHA and BGA, respectively, as compared to control.

Substrate induced respiration allowed us to examine the metabolically active portion of the microbial community responding to glucose addition. Bacterial and fungal communities were assayed independently, and both communities exhibited significantly higher substrate induced respiration when soils had been treated with compost with or without biochar (P < 0.05). CM and B1+CM soils exhibited 426% and 346% (fungal) and 88% and 161% (bacterial) increases in respiration compared to the control soil (Table 4). Ratios of metabolically active fungi: bacteria were also evaluated based on the SIR selective inhibition data. Results indicated the highest FB ratios were observed in the CM (1.30 ± 0.05) and B1+CM (1.30 ± 0.05), which were significantly higher than those of the control, B1 and B2 soils (Table 4). Bacteria were the main drivers of soil respiration in control and biochar treatments, indicated by higher SIR when fungi were inhibited than when bacteria were inhibited (Two-tailed T-tests, P < 0.05). In compost treated soils, the fungi played a larger role in SIR than did the bacteria (Two-tailed T-tests, P < 0.05).

**Table 4:** The substrate induced respiration (selective inhibition).

Treatments	Fungi	Bacteria	FB Ratio	*IAR	% Inhibition
C	341±30 c	447±26 c	0.76±0.09 c	1.00±0.06 a	57±4 b
B1	368±43 c	509±56 c	0.72±0.03 c	0.98±0.01 a	61±19 ab
B2	433±42 c	625±50 bc	0.69±0.07 c	1.00±0.06 a	72±17 ab
CM	1795±121 a	843±82 b	2.13±0.13 a	1.01±0.13 a	96±19 a
B1+CM	1522±182 b	1167±158 a	1.30±0.05 b	1.05±0.10 a	74±8 ab
P	0.000	0.001	0.000	0.755	0.023

*Inhibitor Additivity Ratio.

Treatments include unamended control (CK), 12.5 t ha^-1^ biochar (B1), 25 t ha^-1^ biochar (B2), 5 centimeter (cm) green waste compost (CM), and 12.5 t ha^-1^ biochar and 5 cm compost (B1+CM). Different letters indicate values that are significantly different across treatments (Holm-Šídák, P ≤ 0.05).

**Fig 5:**
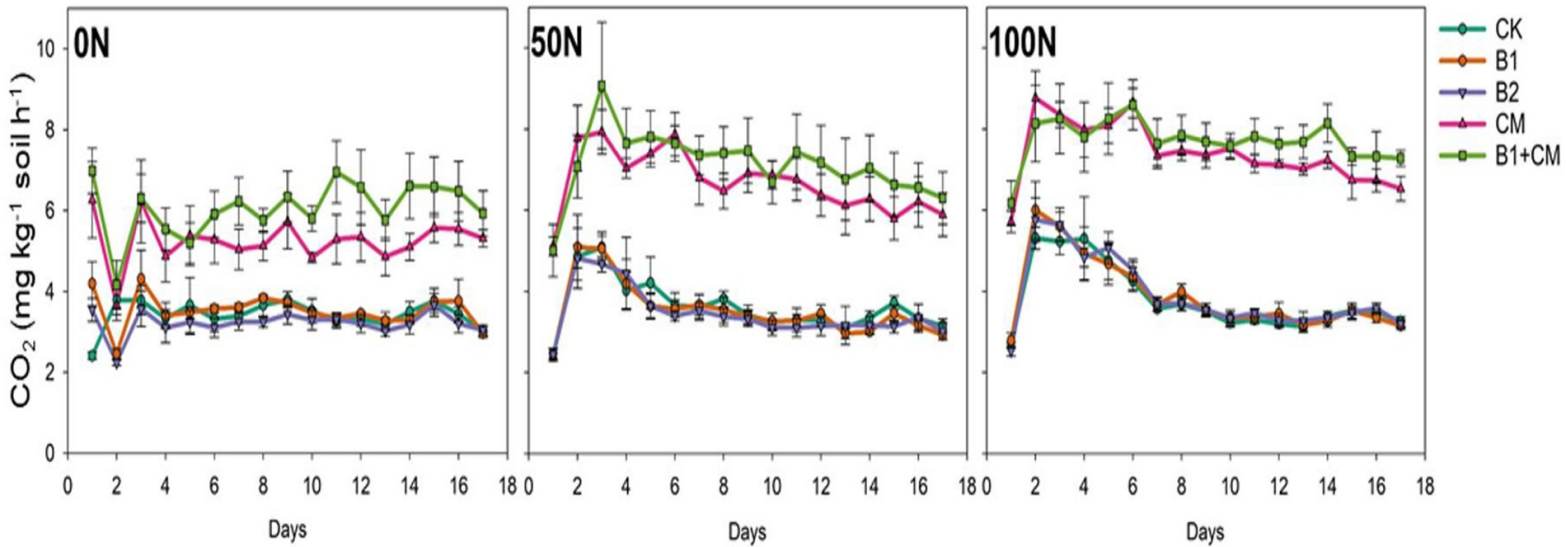
Nitrogen enhanced substrate induced respiration in soils from turfgrass plots with 0N, 50N, and 100N added via urea. Soil treatments included unamended control (CK), 12.5 t ha^-1^ biochar (B1), 25 t ha^-1^ biochar (B2), compost (CM), and 12.5 t ha^-1^ biochar with compost (B1+CM).

To evaluate how communities in the amended soils respond to N fertilization, urea stimulated SIR assays were conducted with two rates of N. In all soils higher SIR rates were achieved with the 50N and 100N compared to 0N treatment (Fig 5). Both rates of N addition stimulated higher respiration rates and prolonged durations of higher SIR in the compost amended soils with and without biochar compared to the control soil (ANOVA, Holm-Sidak post hoc P < 0.05). In comparison with N-stimulated SIR in the control soils, neither B1 nor B2 soils exhibited greater enhanced SIR in response to either N rate.

### Microbial community correlations with edaphic factors

**Fig 6:**
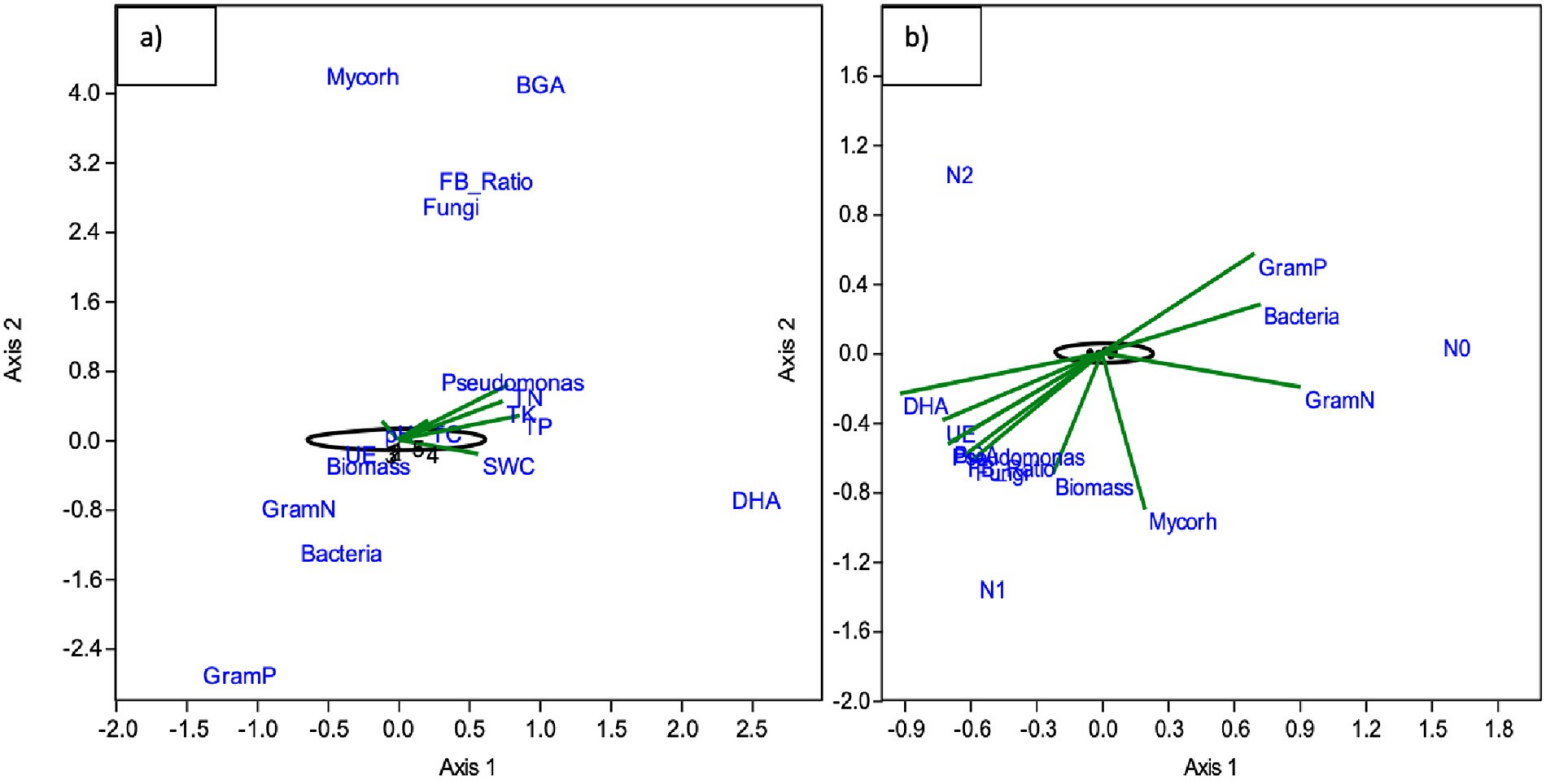
Canonical correspondence analyses revealed associations between soil physical, chemical, and biological properties in turfgrass soils with and without compost and biochar amendments. (a) compares soil properties, PLFA-based microbial groups and enzyme activities (b) compares PLFA-based microbial groups, enzyme activity and nitrogen stimulated-substrate induced respiration (SIR).

A CCA analysis of PLFA-based microbial groups, enzyme activities, and soil nutrient and water data revealed TN to positively correlate with mycorrhizae and total fungi. Total N, TP, and TK had positive correlations with DHA and BGA activity (Fig 6a). Also, the Gram+, Gram-, and total bacterial biomasses were associated with 0N enhanced SIR, while Mycorrhiza, *Pseudomonas*, total biomass, and DHA were all correlated with 50N enhanced SIR (Fig 6b). Mantel tests and Random Forest analyses revealed factors that corresponded with the 16S rRNA-based bacterial/archaeal community profiles. Enzymatic activity, TP, TK, SIR selective inhibition, and respiration rates without N stimulus were strongly correlated with community composition (Table 5). To identify which bacterial/archaeal taxa were the most important in delineating the organic amendments, treatments were grouped as follows; has biochar (B1, B2 and B1+CM) and has compost (CM and B1+CM). A Random Forest analysis was employed to identify which classes were important to differentiating the communities in these groups versus the other treatments and control unamended soils. Overall, the Random Forest results indicated that the compost treatment had more distinguishing taxa (i.e. could be better predicted by the bacterial/ archaeal community class-level profiles) than did biochar (Out of bag error rates of 0% and 45%, respectively). The top three classes with the greatest capacities to distinguish the compost treatments included *Spirochaetia*, *Nitrospira*, and *Anaerolineae* (MSE% > 2.8%). The top three classes important in differentiating soils with and without the biochar amendment included *Nitrosphaerales*, *Deinococci*, and *Acidobacteria*_Gp17 (MSE% > 0.8%). Abundant classes that were important in differentiating organic amendments from one another and the control are signified in Fig 3B (MSE% were in upper 25% ranges).

**Table 5:** Mantel tests of person’s correlations and Random Forest analyses reveal significant associations between 16S rRNA-based community composition and soil biological and chemical properties.

Variables	r Mantel Person	p Mantel Person	R.F. % var explained
**UA**	0.50	**0.001**	**57.15**
**DHA**	0.58	**0.001**	**67.46**
**BGA**	0.47	**0.001**	**58.71**
**pH**	0.06	0.205	-0.58
**TC**	0.042	0.241	-10.64
**TN**	0.19	**0.016**	15.21
**TP**	0.55	**0.001**	**68.7**
**TK**	0.47	**0.001**	**51.47**
**SWC**	0.05	0.221	-6.02
**SRI Bacterial**	0.43	**0.001**	**46.81**
**SRI Fungal**	0.65	**0.001**	**74.49**
**SIR FB Ratio**	0.52	**0.001**	**55.86**
**N50 SIR**	0.08	0.143	9.98
**N100 SIR**	0.12	0.074	13.07
**N0 SIR**	0.39	**0.001**	**46.13**

Mantel correlations are based on Person correlation coefficients. These were also determined based on Spearman correlation, which yielded comparable results. P values < 0.05 and variables with higher than 30% variance explained by the OTU table are in bold text.

## Discussion

### Biochar and compost affect soil properties

After 6 months in the soil, biochar significantly reduced total soil pH compared to the unamended control soil, likely owing to the lower pH of the biochar compared to the soil. Also, biochar can be oxidized in soils (largely on the char surfaces) by chemical and biological activity, which results in a change in the soil pH [45, 46]. The slow oxidization of biochar in soils can generate carboxylic functional groups [45, 47]. The formation of the acidic functional groups can neutralize alkalinity and ultimately reduce the soil pH. In comparison to biochar, compost application significantly altered the soil moisture content, which could be attributed to the improvement in the physical properties of the soil such as (increase in porosity, active surface sites and improvement of soil structure) caused by the addition of organic matter, which favors the water holding capacity of the soil [48]. Correspondingly, compost amendments generally induce beneficial effects on plant available water and other soil properties e.g., soil water retention and plant available water capacity are largely dependent on soil structure (or pore-size distribution), texture (or particle-size distribution), bulk density and TC content [49]. In addition, biochar’s pyrolyzed from different feedstocks vary in the proportion of P and K, and as such, play variable roles in soil P and K fertility levels [50]. A meta-analysis revealed that biochar application in field trials greatly enhanced soil P contents over short term experiments [51]. However, we found a contrasting effect with biochar and found no differences after 6 months of the biochar application on the TN, TP and TK. Compost, on the other hand, did not significantly impact soil pH or water holding capacity, but did significantly enhance TC, TN, TP and TK. The increase in these contents can be attributed to the high nutrient content of the green waste compost. Specifically, the compost contained higher P and K contents than biochar, which corresponds to the enhanced soil TP and TK at the time of the sampling.

### Microbial community composition but not biomass shifted in response to compost and biochar

Microbial biomass was not enhanced in the biochar and/or compost treatments compared to the unamended control. The lack of effect of the treatments on soil biomass six months after their addition is not surprising given the long term, high intensity, carbon input from the turf, which typically provides 1 t ha-1 year-1 of relatively labile carbon as well as a continual daily input of highly labile root exudates [52, 53]. In addition, biochar did not enhance total soil N or NO_3_-N. This indicates that the biochar did not contain appreciable quantities of these nutrients at the rate applied, or was otherwise not as effective at improving microbial nutrient cycling processes and soil nutrient availability compared to previously studied biochar prepared from other feedstocks [54, 55]. Likewise, Domene et al. [56] found no effects of biochar on microbial biomass and respiration activity in corn field plots three years after biochar application at rates less than 30 t ha^1^, and microbial biomass did not increase until biochar was added at the highest rate of 30 t ha^1^.

Random Forest analysis revealed that compost had the strongest treatment effects on microbial community composition and activity, such that the association between community structure and the presence or absence of a soil amendment was greater for compost than for biochar. Enzymatic assays also revealed the highest rates of microbial activity in the compost amended soils, regardless of biochar co-amendment. In the compost amended soils, fungi had higher respiration rates in response to substrate addition than did bacteria, even though fungi and mycorrhizae total abundances and percentages of total biomass were consistent across treatments. A possible conclusion is that compost served as an inoculum or stimulator of fungal populations that were not actively growing during the field soil sampling, and that these fungal communities responded to the input of glucose with high respiration rates. Compost amendment also shaped the bacterial community and microbial classes that were important in differentiating compost amended soils, *Alphaproteobacteria* and *Actinobacteria*, are well known for their roles in organic matter decomposition.

Interestingly, the two rates of applied biochar had differing impacts on microbial community characteristics, indicating both inhibitory and stimulatory capacity of biochar in soil. While, biochar amendment had less of an impact on soil nutrients and community composition and activity than compost, it nonetheless did affect diversity, with significantly reduced Shannon indices in the low biochar treatment compared to the unamended control. Previous research may explain why the lower biochar rate had a greater impact on bacterial/ archaeal taxonomic diversity than the higher application rate. For example, a number of studies indicated that biochar could contain organic pyrolytic byproducts, phenolics and polyphenolics; i.e. compounds that might inhibit soil microbes [4]. The lower rate of biochar may have shown some inhibitory influences on the community diversity. But, as has been demonstrated in prior work, biochar can also supply some initially available labile C, which can stimulate the microbial community [57], so the higher rate of biochar and biochar co-applied with compost may have overcame possible detrimental effects from inhibitory compounds present in biochar. However, the influence of biochar on bacterial communities might change with equilibration to the soil environment [45, 46, 58] so these impacts may not be sustained.

Soil pH is one of the main drivers of soil microbial community shifts [59], and biochar has been shown to significantly impact soil pH [60–62]. We found that biochar significantly reduced soil pH, but soil pH was not identified as a factor correlated with bacterial/archaeal community composition or PLFA-based microbial groups. Rather, TN, TP, and TK correlated with bacterial/archaeal community structure and TN correlated with mycorrhizae abundance. *Pseudomonas* biomass decreased with biochar application as compared to control, which was confirmed with both PLFA and sequencing data. Many *Pseudomonas* genera are root-associated and have plant growth promoting and/ or copiotrophic characteristics [63, 64]. The relative abundances of *Alphaproteobacteria* and *Acidobacteria* classes significantly shifted with biochar or compost addition. Lower ratios of *Proteobacteria* or *Alphaproteobacteria*: *Acidobacteria* have been associated with reduced tropic statuses in soils [64–66] (and both ratios were significantly lower in biochar amended soil without compost compared to the control). The relatively low pH of the biochar, and corresponding shift in total soil pH also may have promoted *Acidobacteria* populations, in contrast to previous findings for forest soils [1, 67].

At the phylum/class levels, *Chloroflexia*/*Chloroflexi* were one of the few taxa to have higher relative abundances in response to both biochar and compost addition. Zhang et al. [68] revealed that the prevalence of CO_2_ fixing microbes belonging to these taxa can enhance soil C sink functions. Soil C was significantly enhanced in the B2, CM, and B1+CM treatments. Hence, future investigations evaluating C sink potentials associated with these amendments should consider soil C contributions that are directly from the feedstock and those attributed to altered community function. Bacteria belonging to the *Nitrospirae* phylum are involved in nitrification and had reduced relative abundances in soils amended with biochar, compost, or both biochar and compost. Reduced populations of nitrifying bacteria could be explained by increased sorption and lower availability of nitrogen as a substrate for nitrifying bacteria and subsequent N loss via denitrification. Furthermore, the class and phylum *Gemmatimonadetes* increased in relative abundances in response to both biochar and compost addition and this class was a distinguishing taxon in compost amended soils. Members of the *Gemmatimonadetes* phylum are associated with arid soils and have been suggested to be adapted to low soil moisture [69]. This decline indicates that the variation in the soil moisture with the organic soil amendments likely impacted the community structure.

### Amendments impacted microbial activity

Extracellular enzymes are key in soil C and N cycling processes, and the potential enzyme activities have been used for decades as indicators of soil health and nutrient turnover [70]. Enzyme activity responds quickly to the addition of organic or inorganic material in the soil [71]. For example, DHA and BGA activities are consistently enhanced with supplementation [72, 73]. Our results revealed that UA, DHA, and BGA enzyme activities were not affected by biochar application alone but adding compost greatly enhanced their activity. While many studies have shown enhanced enzymatic activity in response to biochar [74, 75], these results were typically collected within 100 days of biochar application. The 6-month period between biochar application and soil enzyme analyses conducted in this experiment may have contributed to a lack of significant impact of biochar alone. This effect may be explained by how biochar and compost affected key soil nutrients; biochar altered soil physical and chemical properties by changing the soil pH and SWC, but compost supplied essential nutrients (N, P, K) to microbes, so the legacy impact of the amendments on soil enzymes will likely be different. The composted material also had greater dissolved carbon which could have enhanced microbial enzyme activity. Enzymes may also absorb on the surface of biochar, which can impact enzymatic process rates and reduce carbon mineralization [70].

Urea fertilization is a common practice in turfgrass management and the stability of organic amendments may be altered as soil N supplies are enriched. We evaluated the stimulatory effect of added N on substrate induced respiration to evaluate soil microbial activity with reduced-to-no N limitations, and found that microbial decomposers quickly respond to labile carbon and nitrogen inputs. Communities in compost-amended samples had a higher response of respiration to the added N. While not statistically significant, communities in biochar amended soils did have consistently higher respiration rates after N addition than did those in unamended soils. However, we found no effect of nitrogen addition in biochar treatments, while N additions significantly enhanced CO_2_ in compost amended the soil with and without biochar addition. Accelerated CO_2_ emissions in response to N additions have frequently been observed in agricultural soils [76]. Sucrose addition with N significantly accelerated mineralization of native SOM or labile C as reported by Chen et al. [77], whereas mineral N added in biochar treatments was little influenced in the long term. We saw greater enhanced respiration with N stimulus in compost amended soils than in biochar amended soils and unamended controls. This likely indicates a greater recalcitrance of biochar in turf soils. Thus, the benefits conferred from the biochar amendment, such as enhanced soil pH and water holding capacity, may persist for longer durations than those of more biologically available organic amendments like compost.

## Conclusions

These results reveal impacts of both biochar and compost amendments in arid-zone soils, with low clay content, under turfgrass management. Both amendments resulted in shifts in microbial community profiles and activities, but not total biomass. Compost amendment had more significant impacts on soil nutrients, water holding capacity, microbial respiration and enzymatic activity, fungal respiration, and nitrogen-enhanced substrate induced respiration than did biochar amendment. Biochar caused a greater reduction in soil pH and this corresponded with significant increased relative abundances of many *Acidobacteria* classes. Many taxa involved in C fixation, C decomposition, and N cycling were significantly impacted by biochar, compost, and/or their co-application, but compost elicited more significant shifts in microbial profiles. We found that amendment-induced shifts in soil nutrient and carbon contents, pH, and water holding potential were mirrored by shifts in relative abundances of microbial taxa involved in C and N cycling processes. Further, N stimulus of substrate induced respiration was much greater in compost amended soils than biochar-amended or the un-amended control soils. This signifies potential recalcitrance of biochar in soil and suggests that it may confer soil health benefits for longer durations post soil incorporation. Overall, these results can be considered when selecting amendments for soil health management. If a soil is biologically void, has high incidence of plant disease, and or low microbial activity, then the compost with or without biochar may be preferable to stimulate stark community shifts and promote activity. But, in soils for which native soil microbiota are not concerning or even beneficial, biochar may offer means to enhance soil properties without eliciting major compositional shifts. Additional research should validate if shifts in microbial community compositions and activities revealed here-in play significant functional roles in the field.
